# High prevalence of urogenital infection/inflammation in patients with azoospermia does not impede surgical sperm retrieval

**DOI:** 10.1111/and.13401

**Published:** 2019-08-27

**Authors:** Adrian Pilatz, Jill Kilb, Huelya Kaplan, Daniela Fietz, Hamid Hossain, Christian G. Schüttler, Thorsten Diemer, Martin Bergmann, Eugen Domann, Wolfgang Weidner, Florian Wagenlehner, Hans-Christian Schuppe

**Affiliations:** ^1^ Department of Urology Pediatric Urology and Andrology Justus Liebig University Giessen Giessen Germany; ^2^ Hessian Centre of Reproductive Medicine Justus Liebig University Giessen Giessen Germany; ^3^ Department of Trauma, Hand and Reconstructive Surgery Justus Liebig University Giessen Giessen Germany; ^4^ Institute of Veterinary Anatomy, Histology and Embryology Justus Liebig University Giessen Giessen Germany; ^5^ Institute for Medical Microbiology Justus Liebig University Giessen Giessen Germany; ^6^ Institute for Laboratory Medicine and Microbiology Klinikum St. Marien Amberg Amberg Germany; ^7^ Institute for Medical Virology National Reference Laboratory (NRZ) for HBV and HDV Justus Liebig University Giessen Giessen Germany

**Keywords:** azoospermia, genital tract inflammation, infertility, male accessory gland infection, TESE

## Abstract

Considering infection/inflammation to be an important risk factor in male infertility, the aim of this study was to make a comprehensive evaluation of the prevalence of urogenital tract infection/inflammation and its potential impact on sperm retrieval in azoospermic patients. In this prospective study, 71 patients with azoospermia were subjected to an extensive andrological workup including comprehensive microbiological diagnostics (2‐glass test, semen, testicular swab and testicular tissue analysis) and testicular biopsy/testicular sperm extraction (TESE). Medical history suggested urogenital tract infection/inflammation in 7% of patients, 11% harboured STIs, 14% showed significant bacteriospermia, 15% had seminal inflammation, 17% fulfilled the MAGI definition, and 27% had relevant pathogens. At the testicular level, 1 patient had a swab positive for bacteria, no viruses were detected, tissue specimens never indicated pathogens, whereas histopathology revealed focal immune cell infiltrates in 23% of samples. Testicular sperm retrieval rate was 100% in obstructive and 46% in nonobstructive azoospermia. None of the infection/inflammation‐related variables was associated with the success of sperm retrieval or inflammatory lesions in the testis. The high prevalence of urogenital infection/inflammation among azoospermic men underpins their role as significant aetiologic factors in male infertility. However, this observation does not refer to the chances of sperm retrieval at the time of surgery/TESE.

## 1 INTRODUCTION

Urogenital infections and inflammation are accepted as significant aetiologic factors in male infertility (Fijak et al., 2018; Gimenes et al., 2014; Jungwirth et al., 2012; Weng et al., 2014). Although the available data are extremely heterogeneous, between 6% and 44% of all male cases with infertility are reported to be of infectious/inflammatory origin (Ahmed, Bello, Mbibu, Maitama, & Kalayi, 2010; Bayasgalan, Naranbat, Radnaabazar, Lhagvasuren, & Rowe, 2004; Comhaire, De Kretser, Farley, & Rowe, 1987).

In patients with obstructive azoospermia (OA), the number is even higher, with 22%–47% of all patients having an infectious aetiology (Chan, Brandell, & Goldstein, 2005; Han, Liu, Zhou, Tian, & Zhang, 2016). This is plausible since bacterial ascension through the seminal tract is common and generally found in patients suffering from acute symptomatic epididymitis (Pilatz et al., 2015). In these patients, post‐infectious azoospermia is evident in up to 10% of cases (Rusz et al., 2012) and suspected to be related to epididymal obstruction (Gao & Wang, 2016).

In patients with nonobstructive azoospermia (NOA), bacterial ascension to the testes in terms of epididymo‐orchitis (Pilatz et al., 2015) or haematogenous spread mainly by viruses can be presumed (Dejucq & Jegou, 2001). In this connection, impaired spermatogenesis and infertility after acute epididymo‐orchitis or mumps orchitis have been reported and histologically confirmed (Osegbe, 1991; Zhang et al., 2015). However, the majority of men seeking consultation for couple infertility and suffering from azoospermia are asymptomatic and never report an episode of acute genital tract infection/inflammation (Jungwirth et al., 2012; Schuppe et al., 2017). Consequently, it is more common to describe infectious and/or inflammatory conditions of the male genital tract as “male accessory gland infection” (MAGI) (Comhaire, Verschraegen, & Vermeulen, 1980; Rowe, Comhaire, Hargreave, & Mahmoud, 2000). As the diagnosis is primarily based on abnormalities in the ejaculate, organ‐specific attribution of infectious/inflammatory signs remains difficult (Schuppe et al., 2008). Thus, patient categorisation using MAGI criteria does not necessarily reflect testicular or epididymal inflammation and its sequelae (Fijak et al., 2018).

Notably, in asymptomatic azoospermic patients undergoing testicular sperm extraction (TESE), histological analysis reveals focal immune cell infiltrates in the interstitial compartment in about 30% of cases (Chen, Duan, Haidl, & Allam, 2016; Fijak et al., 2018). However, only a few studies investigated pathogens in testicular tissues and provided suggestive evidence that the testis is not sterile (Alfano et al., 2018; Erles et al., 2001; Martorell et al., 2005; Sripada et al., 2010). In addition, it should be considered that various bacterial and viral species with undefined pathogenicity are detected in the semen of healthy males and patients presenting with sub/infertility (Mandar, Turk, Korrovits, Ausmees, & Punab, 2018; Neofytou, Sourvinos, Asmarianaki, Spandidos, & Makrigiannakis, 2009).

Considering infection/inflammation to be an important risk factor in male infertility and the frequent presence of pathogens within the seminal tract, the aim of this prospective study was to make a comprehensive evaluation of the prevalence of urogenital tract infection/inflammation and its potential impact on sperm retrieval in azoospermic patients undergoing testicular biopsy/TESE.

## 2 MATERIALS AND METHODS

### 2.1 Ethical approval

The prospective study was approved by the local institutional review board (Medical Faculty, Justus Liebig University Giessen, Germany, Ref. No. 26/11). All patients gave written informed consent before participating in this study. The study was performed according to the Declaration of Helsinki.

### 2.2 Study population

From May 2011 to May 2014, a total of 105 infertile men with repeatedly confirmed azoospermia were assessed. The study protocol provided for the exclusion of patients with: men with a history of vasectomy, congenital absence of the vas deferens, spermatogenetic arrest, AZF a and b deletions, hypogonadotrophic hypogonadism and ejaculatory disorders and those who declined study participation or surgery. Thus, the study population consisted of 71 patients (Figure S1).

### 2.3 Andrological investigations

All patients were subjected to an extensive andrological workup including structured assessment of past medical history including questionnaires (e.g. NIH‐CPSI) to assess prostatitis‐like symptoms (Lotti et al., 2014; Wagenlehner et al., 2013) and physical examination, endocrinological investigations and genetic analysis for karyotype, AZF deletions and CFTR gene mutations (Jungwirth et al., 2012). Ultrasound investigations followed a standardised protocol, as published in detail elsewhere (Lotti & Maggi, 2015; Pezzella et al., 2013; Pilatz, Altinkilic, Kohler, Marconi, & Weidner, 2011; Pilatz, Rusz, Wagenlehner, Weidner, & Altinkilic, 2012).

### 2.4 2‐glass test (urine sampling)

**Figure 1:**
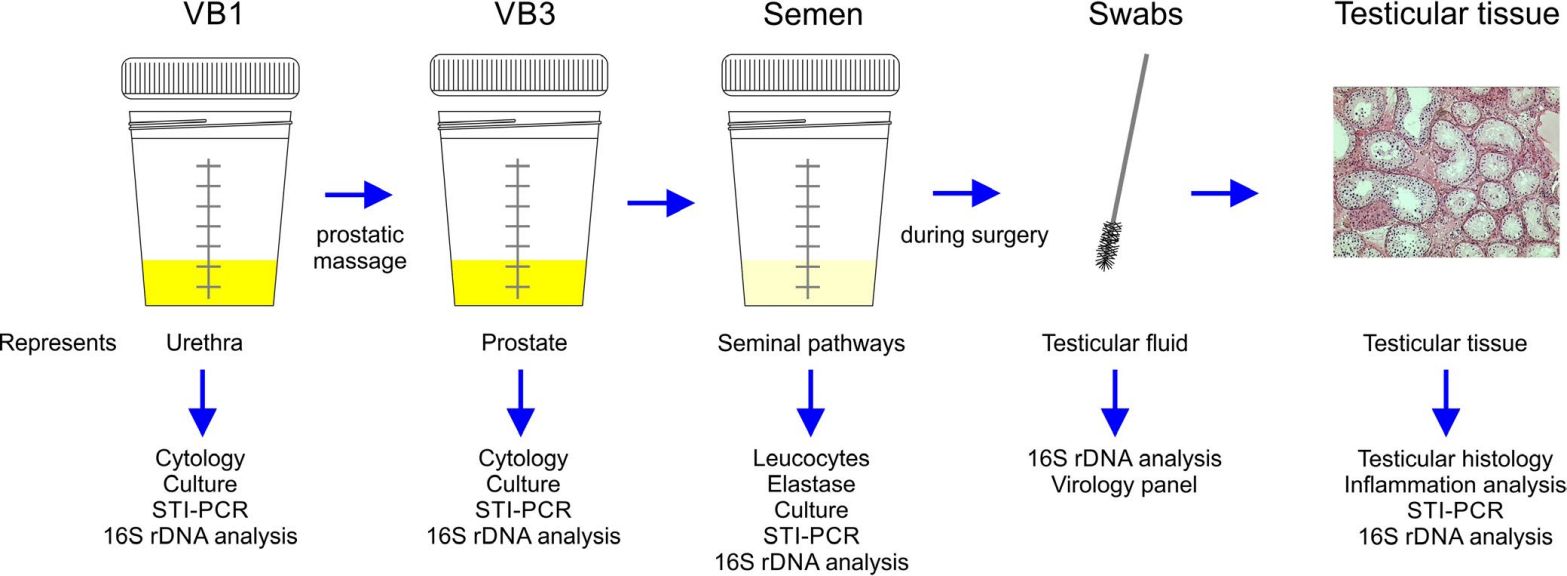
Illustration of diagnostic procedures regarding assessment of infection/inflammation in the urogenital tract

A standardised 2‐glass test consisting of first voided urine (VB1) and post‐prostatic massage urine (VB3) was performed for segmental localisation of pathogens and inflammatory signs within the urogenital tract (Figure 1; Nickel et al., 2006). Patients had been instructed to clean foreskin and glans before voiding and to limit sample volumes to 5 ml. In addition to the microbiologic diagnostics, urine specimens were tested for the presence of leucocytes, using a counting chamber to analyse the centrifuged sediment of 4 ml urine (centrifugation at 100 × *g* for 5 min) after eosin/azure staining according to the manufacturer's protocol (Hemacolor; Merck). A cut‐off value of ≥20 leucocytes per ml in VB3 was considered to be indicative for prostatitis if VB1 was free of leucocytes (Wagenlehner et al., 2013).

### 2.5 Semen analysis

Semen samples were collected by masturbation into a sterile container at the clinic directly after the 2‐glass procedure (Figure 1). Men had been asked to adhere to a sexual abstinence of 2–7 days. Semen analysis was performed within 1 hr of collection according to WHO (2010) recommendations. Confirmation of azoospermia referred to 1 ml aliquots of semen centrifuged at 3,000 × *g* for 15 min. As part of standard processing, the concentration of peroxidase‐positive leucocytes was determined (LeucoScreen; FertiPro). In addition, polymorphonuclear (PMN) elastase reflecting local inflammation was measured in cell‐free seminal plasma by means of an enzyme‐linked immunosorbent assay in each semen sample (Demeditec Diagnostics GmbH). Levels of neutral α‐glucosidase and fructose (total enzymatic activity) at neutral pH were determined by spectrophotometrical methods (Ludwig et al., 2001). Zinc was assessed using a commercially available kit (Zinc Kit; Bako). From each native semen sample, 100 µl was used for the comprehensive microbiological workup. Relevant bacteriospermia was diagnosed for common urinary tract pathogens with ≥10^3^ CFU/ml (Schuppe et al., 2017).

### 2.6 Testicular swabs and sperm retrieval

All patients underwent a combined trifocal/micro‐TESE by two experienced microsurgeons, as detailed before (Marconi et al., 2012). In eight cases with intra‐operative suspicion for obstruction, the procedure was extended by additional microscopic epididymal sperm aspiration (MESA). From each incision, specimens for cryopreservation and histology were harvested.

During surgery, testicular swabs were taken from each TESE and M‐TESE incision to collect testicular fluid using sterile and DNA‐free swabs (ESwab; Copan). As intra‐individual controls, initial swabs were taken from the intact tunica albuginea. A small testicular sample of each patient was used for future microbiological investigations. All material was immediately stored at −80°C until further analysis (see below).

### 2.7 Microbiological diagnostics

Extensive microbiological diagnostics were performed in all samples (Figure 1). Microbiological evaluation in urine (VB1 and VB3) and semen samples included standard culture and species‐specific PCR for sexually transmitted diseases (STI‐PCR). In the case of no bacterial growth and negative STI‐PCR, specimens were further investigated for the presence of bacterial 16S rDNA. Comparably, testicular swabs and testicular tissue specimens underwent STI‐PCR and 16S rDNA analyses.

To assess the bacterial count, 1 and 10 µl of the urinary specimen were inoculated on CLED agar plates (Oxoid Deutschland GmbH) with calibrated plastic loops. For bacteriological and fungal culture, urine specimens were inoculated on MacConkey, 5% sheep blood and Sabouraud agar plates (Oxoid Deutschland GmbH) with a 0.01 ml plastic loop and incubated at 37°C for 24 hr under aerobic conditions. The cultured bacteria were identified by MALDI‐TOF technology (matrix‐assisted laser desorption/ionisation time‐of‐flight) using the VITEK^®^ MS according to the manufacturer's instructions (bioMérieux).

Chromosomal DNA was extracted from urine and semen samples using the Maxwell^®^ 16 Tissue DNA Purification kit (Promega GmbH) on the automated Maxwell^®^ 16 MDx Instrument, Type AS3000 (Promega GmbH) according to the manufacturer's recommendations. Chromosomal DNA from bacteria was extracted from testicular swabs and tissues using the QIAamp DNA Investigator kit (Qiagen). The purified DNA was used directly for STI‐PCR targeting *Mycoplasma genitalium*, *Mycoplasma hominis*,* Ureaplasma urealyticum*,* Chlamydia trachomatis* and *Neisseria gonorrhoeae* in the hyplex^®^ STD system (AmplexBioSystems GmbH) according to the manufacturer's recommendations.

With regard to negative cultures, STI‐PCR in urine and semen samples, and all testicular swabs and tissues, the previously purified DNA was subjected to broad‐range 16S rDNA‐PCR for detection of bacterial DNA. Amplification and detection of the 16S rDNA was performed using the forward primer 0933F and the reverse primer 1407R, as described previously (Domann et al., 2003). The PCR products were analysed using denaturing high‐performance liquid chromatography (DHPLC) on the WAVE^®^ 3500 DNA Fragment Analysis System (Transgenomic), as described previously (Domann et al., 2003; Imirzalioglu, Hain, Chakraborty, & Domann, 2008; Imirzalioglu et al., 2014). Sequences of the 16S rDNA genes were aligned using the Clustal method from MegAlign (DNASTAR Inc.). The nucleic acid sequences obtained were analysed using the algorithm BLAST at the National Center for Biotechnology Information (NCBI) (http://www.ncbi.nlm.nih.gov/) and the GOLD genomes online database (http://www.genomesonline.org/cgi-bin/GOLD/index.cgi).

### 2.8 Virology

A comprehensive viral analysis was performed in swabs derived from the testicular fluid of the mid‐biopsy position of the testis. Viral investigations based on detection by PCR using a panel (Luminex 5N01‐02) included influenza viruses A and B, human parainfluenza viruses type 1–4, respiratory syncytial virus, human adenoviruses, human coronaviruses (229E, OC43, NL63, HKU1), human entero‐ and rhinoviruses, human bocavirus and human metapneumovirus. Single PCRs were performed for herpes simplex virus 1 and 2, varicella zoster virus VZV (Qiagen 4500065) and mumps virus (FTD 13‐48/6).

### 2.9 Testicular histology

For histological evaluation, specimens from each testicular incision site were immediately fixed in Bouin's solution and processed according to the routine protocols. Histopathologic workup included assessment of spermatogenesis and systematic identification of testicular inflammatory lesions. The semi‐quantitative score count evaluation of spermatogenesis was performed according to Bergmann and Kliesch (Bergmann & Kliesch, 2010): For each individual retrieval site, the number of tubules containing elongated spermatids is divided by the total number of tubules examined × 10; hence, score values range from 0 to 10. The overall histological diagnoses were classified into four groups: normal spermatogenesis (score 8–10), hypospermatogenesis (1–7), predominant tubular atrophy (0.1–0.9) and Sertoli cell‐only tubules (SCO) (0), as reported by Bergmann and Kliesch (2010). Patients with maturation arrest (0) were excluded from the study. Sperm retrieval was considered successful if a score value was >0 for any position and patient.

Testicular inflammation was systematically evaluated in all biopsy specimens using HE sections (Bergmann & Kliesch, 2010; Schuppe et al., 2008). Detection of inflammatory infiltrates with at least one focus in any site per patient was defined as a positive result. These focal infiltrates mainly contained lymphocytes in a peritubular distribution and were considered as significant inflammatory lesions (Klein et al., 2016; Figure [Link], [Link], [Link]).

### 2.10 Statistical analysis

Data are expressed as median and inter‐quartile range (IQR) in the case of metric variables and number (%) when having nominal/categorical variables. Statistical analysis was done to investigate possible associations between different variables associated with urogenital tract inflammation/infection and the outcome of testicular sperm retrieval, testicular inflammation and other clinical parameters. With regard to the categorical variables, the Fisher exact test was applied when comparing two categories, while the chi‐square test was used for more than two categories. The Mann–Whitney *U* test was used for metric variables in two groups, while the Kruskal–Wallis test was applied in three groups. A value of *p* < .05 was considered statistically significant. Statistical analyses were performed by means of IBM SPSS Statistics 25 for Windows (IBM GmbH).

## 3 RESULTS

### 3.1 Demographics and andrological findings

Complete patient's demographics are given in Table 1. Clinical risk factors for azoospermia are presented in Table 2. Table 3 displays the andrological findings in detail.

**Table 1:** Demographics of the study population

Parameter	Median (IQR), or *n* (%)
Patient's age (years)	34 (30–38)
Body size (cm)	180 (175–184)
BMI (kg/m^2^)	26.0 (23.6–29.4)
Body weight (kg)	82 (75–95)
Age of female partner (years)	30 (27–34)
Sexually active	71 (100%)
Current female partner	71 (100%)
Duration of current partnership (years)	7 (4–10)
Married	54 (76%)
Duration of marriage (years)	2 (0–3)
Duration of unwanted childlessness (years)	2 (1.5–3)
Number of brothers	1 (0–2)
Number of sisters	1 (0–1)
Lifetime sexual partners^a^	5 (3–10)
History of miscarriage in family	11 (16%)
Involuntary childlessness in family	11 (16%)
Alcohol consumption	Frequently (Rarely–Occasionally)
Previous smokers	17 (24%)
Current smokers	18 (25%)
History of drug abuse (cannabis)	9 (13%)
Current drug abuse (cannabis)	1 (1%)
Exposure to pollutants	15 (21%)
Heat	4 (6%)
Radioactivity	1 (1%)
Solvents	6 (9%)
Multiple	3 (4%)

a Data from *n* = 68 patients.

**Table 2:** Overview of clinical risk factors for azoospermia

Factor	Patients (%)^a^
History of cryptorchidism	16 (22)
Genetic disorders (Klinefelter syndrome, Y chromosome microdeletion [AZFc])	12 (17)
History of cancer	8 (11)
History of urogenital tract infection/inflammation	5 (7)
Unexplained	35 (49)

a Five patients with multiple risk factors.

**Table 3:** Andrological parameters of the study population

Parameter	Median (IQR), or *n* (%)
Semen	
Volume (ml)	2.5 (1.8–3.6)
pH value	7.6 (7.3–7.8)
Sperm concentration (million/ml)	0 (0–0.0)
Peroxidase‐positive leucocytes (million/ml)	0.1 (0–0.1)
Fructose (µmol/ejaculate)	36.8 (19.7–64.2)
Glucosidase (mU/ejaculate)	29.5 (17.7–52.8)
Elastase (ng/ml)^a^	49 (18–153)
Zinc (µmol/ejaculate)^b^	9.5 (6.1–17.4)
Hormones	
FSH (mU/ml)	19.8 (13.8–29.1)
LH (mU/ml)	7.3 (4.3–12.7)
Testosterone (nmol/L)	12.2 (8.8–15.5)
Free testosterone (pmol/L)	241.2 (197.1–296.3)
SHBG (nmol/L)	29.2 (24.4–41.6)
Albumin (g/L)	47.6 (45.9–49.1)
Oestradiol (pmol/L)	110.1 (88.1–135.8)
Prolactin (uIU/ml)	163 (123–201)
Ultrasound	
Total testicular volume (ml)^c^	14.2 (7.9–19.9)
Mean testicular volume (ml)^c^	7.2 (4.1–10.2)
Epididymal head height (mm)^d^	9.8 (7.9–11.6)
Epididymal head thickness (mm)^d^	7.9 (6.8–9.7)
PSV testicular artery (cm/s)^c^	6.8 (5.2–8.3)
PSV intratesticular arteries (cm/s)^c^	4.3 (3.5–5.1)
Right varicocele	0 (0%)
Left varicocele	19 (27%)
Subclinical	12 (17%)
Grade I	2 (3%)
Grade II	2 (3%)
Grade III	3 (4%)

Abbreviation: PSV, peak systolic velocity.

a *n* = 70.

b *n* = 69.

c Six patients with single testis excluded.

d Seven patients with single epididymis excluded.

### 3.2 Cytological and microbiological findings in urine and semen

Urine cytology revealed increased leucocyte numbers in VB3 in only one patient, indicating a questionable inflammatory reaction of the prostate. A leucocyte reaction in VB1 was not detectable. Leucocytospermia was detected in 4 of 71 (6%) and increased elastase levels in 11 of 70 (16%) of patients.

The detailed individual results of the 2‐glass test, semen analysis, testicular swabs and testicular tissue regarding clinically relevant pathogens are presented in Table 4. Common urinary tract pathogens (*Escherichia coli*, *Pseudomonas aeruginosa*, *Enterococcus faecalis*) with relevant numbers (≥10^5^ CFU/ml) were found in three patients (one in each case) whereby the same pathogen was also detected in urinary specimens and semen in two of those patients. Eight patients had significant bacteriospermia (≥10^3^ CFU/ml) with common urinary bacteria without urinary tract infection. There was no evidence of bacterial prostatitis. Sexual transmitted bacteria were detected in 8 of 71 (11%) patients. Here, *Ureaplasma urealyticum* was the dominant pathogen, being present in all seven cases in VB1. In cases where *Chlamydia trachomatis* and *Mycoplasma hominis* were found, the bacteria were present in all urine fractions and in semen.

**Table 4:** Synopsis of clinically relevant pathogens in the urogenital tract

Patient	VB1	VB3	Ejaculate	Testicular swabs	Testicular tissue
2	*Ureaplasma urealyticum*	Ø	Ø	Ø	Ø
6	Ø	Ø	*Escherichia coli* 27.500 CFU/ml	Ø	Ø
7	*Ureaplasma urealyticum*	Ø	Ø	Ø	Ø
10	*Mycoplasma hominis*	*Mycoplasma hominis*	*Mycoplasma hominis*	Ø	Ø
11	*Chlamydia trachomatis*, *Ureaplasma urealyticum*	*Chlamydia trachomatis*, *Ureaplasma urealyticum*	*Chlamydia trachomatis*	Ø	Ø
23	*Pseudomonas aeruginosa* >10^5^ CFU/ml	*Pseudomonas aeruginosa* >10^5^ CFU/ml	*Pseudomonas aeruginosa* >10^5^ CFU/ml	Ø	Ø
24	*Escherichia coli* >10^5^ CFU/ml	*Escherichia coli* >10^5^ CFU/ml	*Escherichia coli* 12.100 CFU/ml	Ø	Ø
28	Ø	Ø	Ø	*Enterobacter cloacae*, *Leclercia adecarboxylata*	Ø
30	Ø	Ø	*Enterococcus faecalis* 46.200 CFU/ml	Ø	Ø
35	*Ureaplasma urealyticum*	Ø	Ø	Ø	Ø
37	Ø	Ø	*Klebsiella oxytica* >10^5^ CFU/ml	Ø	Ø
44	*Ureaplasma urealyticum*	*Ureaplasma urealyticum*	*Ureaplasma urealyticum*	Ø	Ø
48	Ø	Ø	*Escherichia coli* 49.500 CFU/ml	Ø	Ø
63	*Ureaplasma urealyticum*	*Ureaplasma urealyticum*	Ø	Ø	Ø
64	Ø	*Enterococcus faecalis* >10^5^ CFU/ml	Ø	Ø	Ø
69	Ø	Ø	*Enterococcus faecalis* 4.400 CFU/ml	Ø	Ø
77	Ø	Ø	*Enterococcus faecalis* >10^5^ CFU/ml	Ø	Ø
82	*Ureaplasma urealyticum*	*Ureaplasma urealyticum*	*Enterococcus faecalis* 15.400 CFU/ml	Ø	Ø
103	Ø	Ø	*Citrobacter koseri* 5.500 CFU/ml	Ø	Ø

In addition, a high number of noninfectious urethral commensals were detected in numbers up to 10^4^ CFU/ml. The individual patient data regarding the identified commensals are summarised in Table S1.

### 3.3 Microbiological and virological findings in testicular fluid/tissue

In one patient, the evaluation of the 16S rDNA analysis in testicular swabs demonstrated *Enterobacter cloacae* plus *Leclercia adecarboxylata*. The STI‐PCR of the swabs performed in patients with proven STIs in the 2‐glass test was negative in all cases. Finally, 16S rDNA analysis was always negative, especially in testicular tissue specimens with focal inflammatory infiltrates (*n* = 16) and in tissues from patients with significant bacteriuria (*n* = 3) and STIs (*n* = 8) in urine. The virological investigations were always without any virus detection.

### 3.4 Surgical outcome and histopathologic diagnosis

Postoperative infectious complications did not occur in any of the patients.

Successful sperm retrieval was documented in 41 of 71 (58%) patients. Histologically, the following spermatogenetic patterns were recorded: normal spermatogenesis (*n* = 15), hypospermatogenesis (*n* = 9), predominant tubular atrophy (*n* = 17) and SCO (*n* = 30). Of the 15 patients with obstructive azoospermia, eight patients received MESA. In 6 of 8 (75%) cases, the MESA retrieved viable spermatozoa.

Intratesticular inflammatory reactions were found in 16 of 71 (23%) biopsy specimens and categorised as sparse (*n* = 9) or dense (*n* = 7). Figure [Link], [Link], [Link] shows representative inflammatory infiltrates.

### 3.5 Predictors of surgical outcome in terms of sperm retrieval

When evaluating the various infectious/inflammatory parameters with respect to surgical outcome (successful sperm retrieval), no significant associations were found (Table 5). In addition, the presence or absence of testicular focal immune cell infiltrates was not associated with any variable measuring infection/inflammation in the downstream urogenital tract, except for number of lifetime sexual partners (*p* = .037; Table S2).

**Table 5:** Association of parameters indicating infection/inflammation and testicular sperm retrieval

Parameter	OA (*n* = 15)	NOA positive (*n* = 26)	NOA negative (*n* = 30)	*p*
Lifetime sexual partners, median (ICR)^a^	6 (3–15)	5 (3–10)	5 (2–10)	.915
History of urogenital tract infection/inflammation	2/15 (13%)	1/26 (4%)	2/30 (7%)	.517
Amount of leucocytes in VB1 (cells/hpf)^b^	1.0 (0.5–3.5)	0.5 (0.0–1.0)	0.5 (0.0–1.0)	.127
Amount of leucocytes in VB3 (cells/hpf)^b^	0.5 (0.5–1.0)	0.5 (0.0–2.0)	0.5 (0.0–0.5)	.368
Leucocyturia > 20 cells/hpf^c^	1/15 (7%)	0/23 (0%)	0/27 (0%)	.184
Peroxidase‐positive leucocytes, median (ICR) in 10^6^/ml	0.0 (0.0–0.4)	0.1 (0.0–0.1)	0.0 (0.0–0.2)	.957
Leucocytospermia (≥10^6^/ml)	2/15 (13%)	1/26 (4%)	1/30 (3%)	.345
Elastase, median (ICR) in ng/ml^d^	71 (22–264)	47 (28–77)	40 (14–190)	.460
Elastase > 250 ng/ml^d^	4/14 (29%)	1/26 (4%)	6/30 (20%)	.085
Relevant inflammation in urine/semen (leucocytospermia, VB3 > 20 cells/hpf, elastase > 250 ng/ml)	4/15 (27%)	1/26 (4%)	6/30 (20%)	.101
Presence of bacteriospermia (≥10^3^ CFU/ml)	9/15 (60%)	12/26 (46%)	9/30 (30%)	.139
Amount of pathogens in bacteriospermia (CFU/ml)	2,200 (0–15,400)	0 (0–3,300)	0 (0–2,750)	.169
Presence of relevant bacteriospermia^e^	4/15 (27%)	1/26 (4%)	5/30 (17%)	.112
Presence of STIs in urogenital tract	1/15 (7%)	4/26 (15%)	3/30 (10%)	.668
Presence of pathogens >10^5^ CFU/ml in urine specimens	1/15 (7%)	1/26 (4%)	1/30 (3%)	.865
Presence of pathogens in swabs/testicular tissue	1/15 (7%)	0/26 (0%)	0/30 (0%)	.151
All pathogens in urogenital tract	9/15 (60%)	14/26 (54%)	10/30 (33%)	.153
Clinically relevant pathogens in urogenital tract	5/15 (33%)	6/26 (23%)	8/30 (27%)	.775
Fulfilled MAGI definition	6/15 (40%)	1/26 (4%)	5/30 (17%)	**.012**
NIH‐CPSI score total score^f^	3 (2–7)	1 (0–2)	3 (0–8)	**.031**
Focal inflammatory lesions in testicular biopsy specimens (sparse, dense)	4/15 (27%)	8/26 (31%)	4/30 (13%)	.271

Bold values specify significant parameters.

Abbreviation: hpf, high‐power field.

a *n* = 68.

b *n* = 62.

c *n* = 65.

d *n* = 70.

e Urethral commensals (Table S1) excluded.

f *n* = 55.

On the other hand, several well‐known clinical parameters were significantly associated with sperm retrieval (e.g. FSH, LH, testicular volume and epididymal size; Table S3).

## 4 DISCUSSION

In the present study, we comprehensively assessed infection/inflammation‐related parameters in the urogenital tract in patients with azoospermia undergoing testicular biopsy/TESE. Although the medical history suggested previous urogenital tract infection/inflammation in 7% of cases and microbiology revealed relevant bacterial pathogens in 27% of patients, all infection/inflammation‐related variables determined were not associated either with the success of testicular sperm retrieval or with the presence of focal inflammatory lesions in testicular histology seen in 23% of patients.

The reasons for azoospermia are extremely diverse, with about half of the cases related to genetic disorders and cryptorchidism (Tüttelmann et al., 2011). Nevertheless, in a large series consisting of 1583 azoospermic men, 10.3% were categorised as being of infectious aetiology (Tüttelmann et al., 2011). This number compares well with our data. However, the aetiologic category of “infection” in various reports is mainly related to medical history and not based on clinical variables (Chan et al., 2005; Han et al., 2016). To the best of our knowledge, this is the first prospective study which set out to comprehensively evaluate infection and inflammation in patients presenting with azoospermia using a 2‐glass test and analysis of semen, testicular fluid and testicular tissue by measuring inflammatory parameters and performing systematic microbiological investigations. In combination with a systematic clinical workup, this allows a compartment‐specific approach towards genital tract inflammation/infection related to azoospermia.

The rationale for our hypothesis was that experimental studies in animals mimicking epididymitis have clearly demonstrated how ascending pathogens reach the testes within some days and rapidly lead to an impairment of testicular architecture (Fijak et al., 2018; Lu et al., 2013). Comparable observations have been made in a limited number of males (ethical limitations) suffering from acute epididymitis. In the acute phase, a reduction in spermatogenesis and an intratesticular invasion by polymorphonuclear cells were evident in such patients (Osegbe, 1991; Wolin, 1971), while in the follow‐up about 1 year later, disruption of testicular architecture associated with fibrosis, hyalinisation, hypospermatogenesis, maturation arrest or Sertoli cell‐only syndrome was reported (Dietz, 1960; Osegbe, 1991). This shows that genital tract infection is clearly related to impaired testicular architecture and also a possible disruption of the blood–testis barrier (Fijak et al., 2018; Lotti et al., 2018). In addition to testicular damage, epididymal obstruction is frequently a consequence of induced seminal tract infection in animal models (Michel et al., 2016). In humans, there is evidence that azoospermia following acute epididymitis might also be of obstructive origin, since no decrease in testicular volume was detected (Pilatz et al., 2013). Thus, seminal tract obstruction might be a reason for azoospermia. In several case series involving patients with obstructive azoospermia, this is underlined by an infectious aetiology as high as 22%–47% of cases (Chan et al., 2005; Han et al., 2016).

Unlike males suffering from acute urogenital tract infections, most of the affected males counselled for sub/infertility are asymptomatic (Schuppe et al., 2017). Although the MAGI system is commonly used to characterise these patients by clinical and laboratory findings (Comhaire et al., 1980; Rowe et al., 2000; Schuppe et al., 2017), the diagnostics do not consider STI‐PCR testing (Eley & Pacey, 2011), lack soluble inflammatory parameters (e.g. cytokines; Pilatz et al., 2017) and sperm DNA fragmentation (Lotti et al., 2017), and assume a sterile urogenital tract under normal conditions. Recent advances in microbiological techniques, however, have broken with the latter dogma (Hou et al., 2013; Imirzalioglu et al., 2008; Weng et al., 2014). When investigating the seminal plasma microbiome with next‐generation sequencing (NGS), an average number of 135 genera and 569 species were detected in each sample, with *Lactobacillus* (19.9%), *Pseudomonas* (9.85%), *Prevotella* (8.51%) and *Gardnerella* (4.21%) being the most dominant genera (Weng et al., 2014). Interestingly, semen samples with normal parameters were mainly found to be clustered in the *Lactobacillus*‐predominant group (Weng et al., 2014), while another research team failed to demonstrate significant differences between the seminal microbiome of sperm donors and infertile patients (Hou et al., 2013).

The question arises whether the identified microbiome in the semen is really present in the seminal tract or whether the bacteria are just commensals in the distal urethra and skin, which are added to the semen by the process of masturbation. As an immune‐privileged organ due to the blood–testis barrier, testicular tissue is generally believed to be sterile. However, just recently, a microbiome study evaluating testicular tissue from azoospermic and testicular cancer patients revealed that many different bacterial classes can be detected by NGS (Alfano et al., 2018). Of further interest was the fact that the amount of bacterial DNA was increased in patients with NOA compared to those with normal spermatogenesis (Alfano et al., 2018). Finally, no significant differences were identified in NOA patients with positive sperm retrieval (*n* = 5) and those without (*n* = 5; Alfano et al., 2018). Although we did not apply NGS, we were never able to identify any pathogens in testicular swabs and testicular tissue by sensitive 16S rDNA analysis, except for one swab sample which was positive for *Enterobacter cloacae* and *Leclercia adecarboxylata* although the corresponding testicular tissue was negative.

As with bacterial pathogens, data on the role of viruses and their impact on testicular histology are sparse. Older data reported no viral evidence in testicular tissues in patients presenting with acute epididymitis (Wolin, 1971). However, in testicular tissue samples of azoospermic patients undergoing sperm retrieval, adeno‐associated viruses were evident in 10 of 38 biopsies without any statistical differences between viral DNA status and histological diagnosis (Erles et al., 2001). In another TESE series, human papillomaviruses (HPVs) were reported in 12 of 185 (6%) patients with comparable testicular histology undergoing sperm retrieval whereby HPV 16 was the most common genotype (Martorell et al., 2005). One study group working with azoospermic patients (*n* = 52) who had a history of mumps orchitis showed a testicular sperm retrieval rate of 73% (Zhang et al., 2015). To the best of our knowledge, however, we were the first to evaluate such a large viral panel in patients with azoospermia using testicular swabs during TESE, although none of the swabs revealed any viruses.

Immune cell infiltrates are frequently found in testicular biopsies (Chen et al., 2016; Fijak et al., 2018), but their association with spermatogenic failure has yet to be investigated in detail. Our data indicate less inflammatory activity with focal peritubular immune cell infiltrates in patients with Sertoli cell‐only syndrome compared to those patients with positive sperm retrieval. Unfortunately, we failed to show an association between the detected seminal tract pathogens/inflammation and the occurrence of intratesticular inflammatory lesions. Thus, the aetiology of the testicular inflammatory reactions in these patients remains unclear (Fijak et al., 2018).

Our study has some limitation that should be acknowledged: (a) We did not perform NGS on seminal plasma or testicular tissue, as this technique does not allow clinically significant infections of foreign pathogens or contaminants to be dissected. Moreover, at the time of starting the project, reliable NGS was not available. (b) Since we only performed MESA in eight patients in our series and analysed testicular interstitial fluid and testicular tissue directly for pathogens in all patients, we did not examine the MESA fluid separately. (c) Human papillomaviruses were not the subject of virological investigations. (d) Since reproductive medical care was provided separately in cooperating fertility centres, data regarding pregnancy rates or birth rates could not be collected and analysed within the framework of the present study.

## 5 CONCLUSION

The high prevalence of urogenital infections and inflammation among azoospermic men (27%) underpins their role as significant aetiologic factors in male infertility, including obstruction of excurrent ducts and deterioration of spermatogenesis. However, this observation does not refer to the chances of sperm retrieval at the time of surgery/TESE. As injury is likely to have happened far in the past, more specific tools including molecular signatures are required for early noninvasive diagnosis and therapy.

## CONFLICT OF INTEREST

There are no competing interests related to this study.
