# Supplementary figures and images for: High prevalence of urogenital infection/inflammation in patients with azoospermia does not impede surgical sperm retrieval
Source: Andrologia. 2019 Aug 27;51(10):e13401. doi: 10.1111/and.13401 (PMC7147116; doi:10.1111/and.13401)

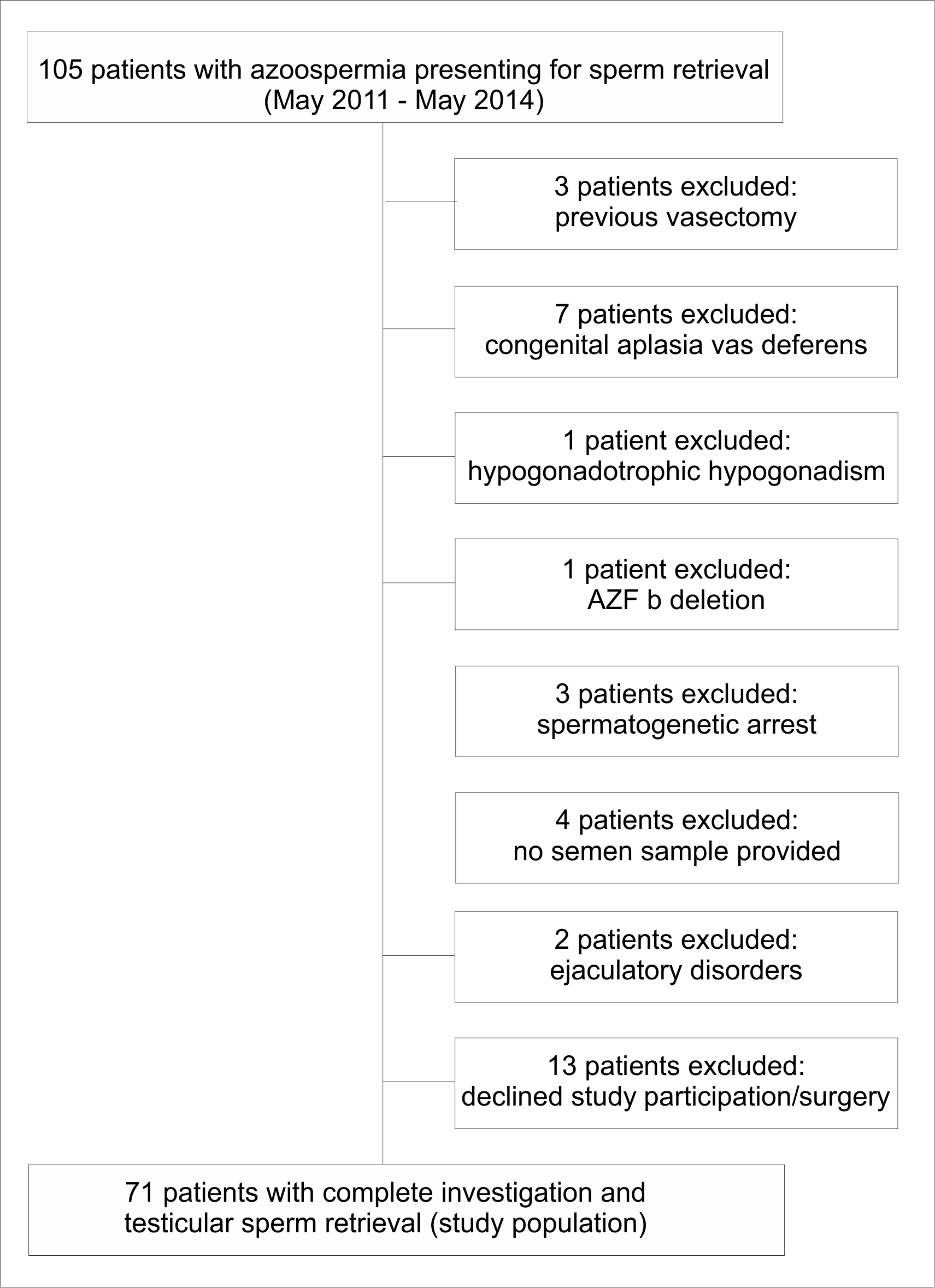

Supplement: Supplementary file 1 [file AND-51-na-s001.tif]

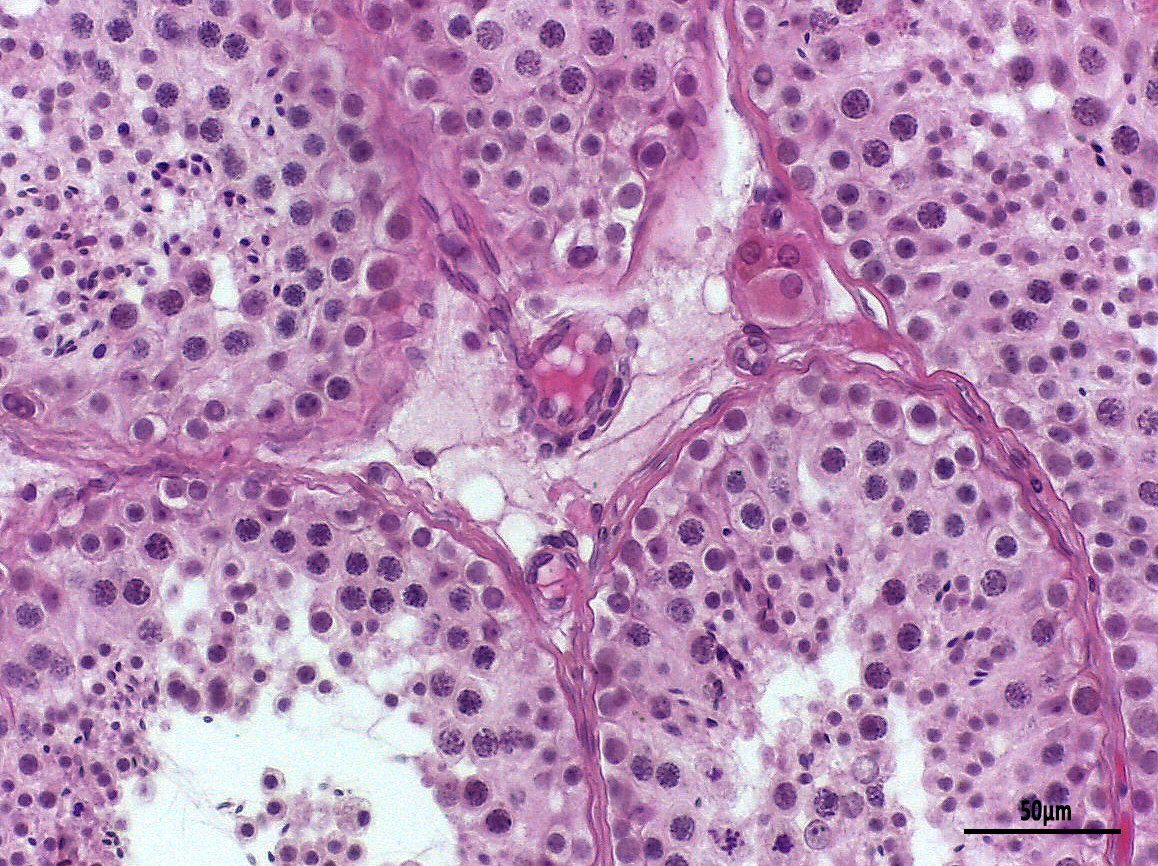

Supplement: Supplementary file 2 [file AND-51-na-s002.tif]

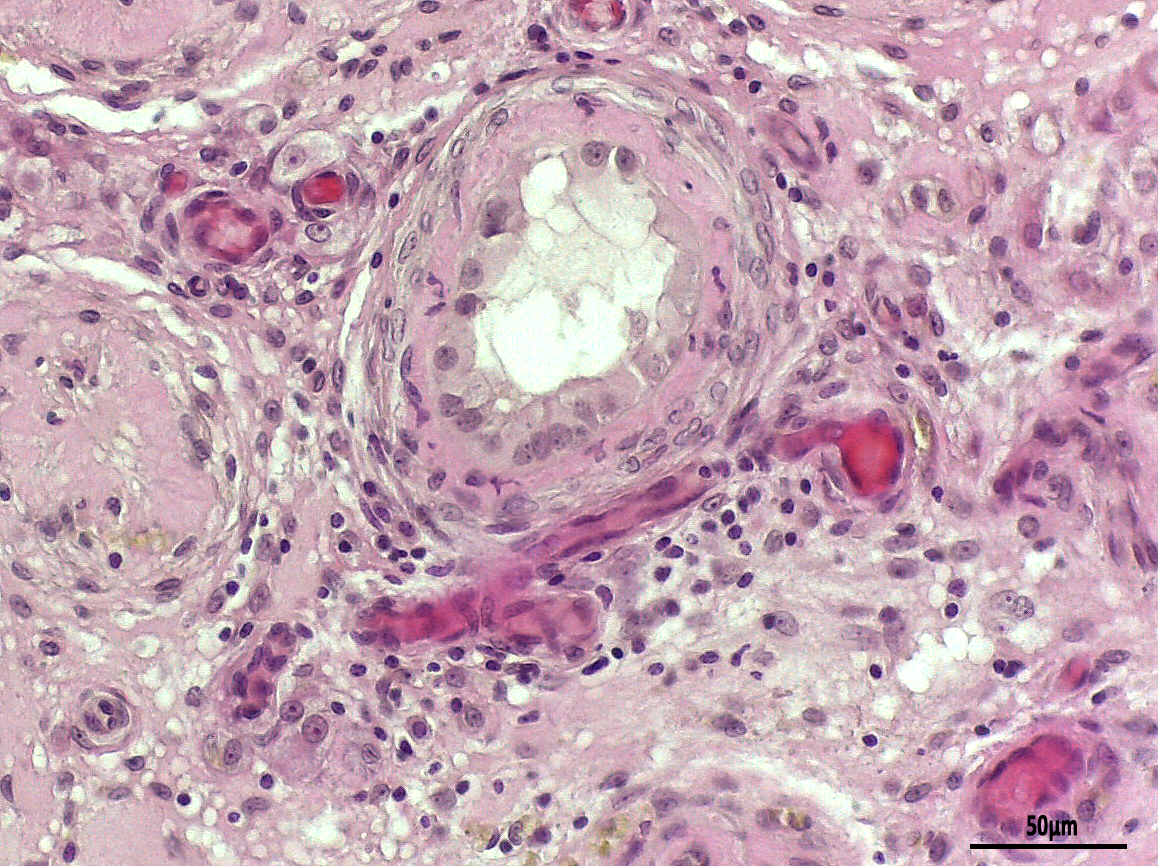

Supplement: Supplementary file 3 [file AND-51-na-s003.tif]

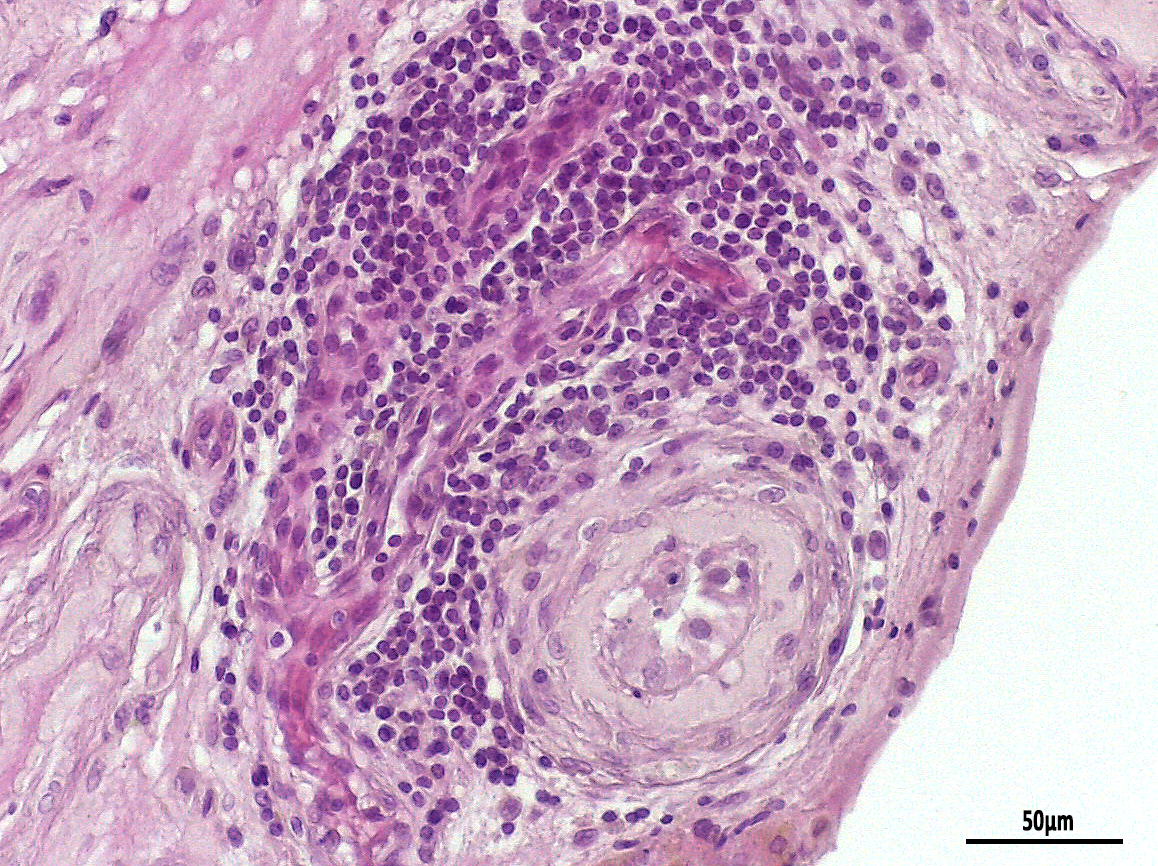

Supplement: Supplementary file 4 [file AND-51-na-s004.tif]
