# Supplementary material for: High prevalence of urogenital infection/inflammation in patients with azoospermia does not impede surgical sperm retrieval
Source: Andrologia. 2019 Aug 27;51(10):e13401. doi: 10.1111/and.13401 (PMC7147116; doi:10.1111/and.13401)
Supplement: Supplementary file 5 [file AND-51-na-s005.docx]

**Supplementary Material**

**Supplementary Figure Legends**

**Supplementary Figure S1** Flowchart of patient inclusion.

**Supplementary Figure S2** Testicular histopathology of azoospermic men: exemplary illustration of the qualitative grading of inflammatory lesions/focal interstitial immune cell infiltration. A) normal spermatogenesis, no infiltrates, B) sparse peritubular immune cell infiltration , C) dense inflammatory infiltrate; B & C depict severe testicular damage with seminiferous tubules containing only Sertoli cells, associated with an altered (thickened) lamina propria, as well as tubular shadows (H&E staining; 200-fold magnification).

**Supplementary Tables**

Supplementary Table SI Synopsis of pathogens considered as urethral commensals

| Patient | VB1 | VB3 | Ejaculate |
| --- | --- | --- | --- |
| 1 | Ø | *Streptococcus mitis* (16S rDNA) | *Streptococcus mitis* (16S rDNA) |
| 2 | *Staphylococcus epidermidis*  <10^3^ CFU/ml | *Staphylococcus epidermidis* <10^3^ CFU/ml | Ø |
| 4 | *Streptococcus* spp. <10^3^ CFU/ml | *Streptococcus* spp. <10^3^ CFU/ml | Ø |
| 8 | Ø | Ø | *Staphylococcus epidermidis* 27.500 CFU/ml |
| 11 | *Staphylococcus epidermidis* <10^3^ CFU/ml | Ø | *Staphylococcus epidermidis* 13.200 CFU/ml |
| 13 | *Staphylococcus epidermidis*  <10^3^ CFU/ml | Ø |  |
| 19 | Ø | *Staphylococcus epidermidis, Streptococcus* spp. <10^4^ CFU/ml | Ø |
| 26 | *Staphylococcus epidermidis & Streptococcus* spp. & *Enterococcus faecalis*  <10^3^ CFU/ml | Ø | *Staphylococcus epidermidis* 1.100 CFU/ml |
| 27 | *Staphylococcus epidermidis & Streptococcus* spp. <10^4^ CFU/ml | Ø | *Streptococcus* spp. 2.200 CFU/ml |
| 28 | *Staphylococcus epidermidis* <10^3^ CFU/ml | *Staphylococcus epidermidis* <10^3^ CFU/ml | Ø |
| 29 | *Staphylococcus epidermidis* <10^3^ CFU/ml | Ø | Ø |
| 33 | *Staphylococcus epidermidis* <10^3^ CFU/ml | Ø | *Staphylococcus epidermidis* 1.100 CFU/ml |
| 34 | Ø | *Streptococcus* spp. <10^3^ CFU/ml | Ø |
| 43 | *Staphylococcus epidermidis & Lactobacillus* spp. <10^3^ CFU/ml | Ø | *Lactobacillus* spp. 3.300 CFU/ml |
| 44 | *Streptococcus* spp. <10^3^ CFU/ml | *Streptococcus* spp. <10^3^ CFU/ml | Ø |
| 46 | *Streptococcus* spp. <10^4^ CFU/ml | *Streptococcus* spp. <10^3^ CFU/ml | Ø |
| 48 | *Escherichia coli & Streptococcus* spp. <10^4^ CFU/ml | *Escherichia coli,* *Streptococcus* spp. <10^3^ CFU/ml | Ø |
| 51 | *Staphylococcus epidermidis* <10^4^ CFU/ml | Ø | *Staphylococcus epidermidis* 1.100 CFU/ml |
| 53 | *Streptococcus* spp. <10^4^ CFU/ml | *Streptococcus* spp. <10^3^ CFU/ml | *Streptococcus* spp. 2.200 CFU/ml |
| 60 | *Staphylococcus epidermidis* <10^3^ CFU/ml | *Staphylococcus epidermidis* <10^3^ CFU/ml | *Staphylococcus epidermidis* 6.600 CFU/ml |
| 62 | *Enterococcus faecalis* <10^4^ CFU/ml | *Enterococcus faecalis* <10^4^ CFU/ml | *Streptococcus* spp. 68.200 CFU/ml |
| 63 | *Staphylococcus epidermidis* <10^3^ CFU/ml | *Staphylococcus epidermidis* <10^3^ CFU/ml | Ø |
| 64 | *Enterococcus faecalis* <10^3^ CFU/ml | Ø | Ø |
| 68 | *Staphylococcus epidermidis* <10^3^ CFU/ml | Ø | Ø |
| 69 | *Streptococcus* spp. <10^4^ CFU/ml | *Streptococcus* spp. <10^4^ CFU/ml | Ø |
| 70 | *Staphylococcus epidermidis* <10^3^ CFU/ml | *Staphylococcus epidermidis* <10^3^ CFU/ml | Ø |
| 73 | *Staphylococcus epidermidis* <10^3^ CFU/ml | *Staphylococcus epidermidis* <10^3^ CFU/ml | *Staphylococcus epidermidis* >10^5^ CFU/ml |
| 75 | *Enterococcus faecalis* <10^4^ CFU/ml | Ø | Ø |
| 77 | *Enterococcus faecalis* <10^4^ CFU/ml | *Enterococcus faecalis* <10^3^ CFU/ml | Ø |
| 78 | *Enterococcus faecalis* <10^4^ CFU/ml | *Enterococcus faecalis* <10^3^ CFU/ml | Ø |
| 80 | Ø | *Staphylococcus epidermidis* <10^3^ CFU/ml | Ø |
| 81 | *Staphylococcus epidermidis & Streptococcus* spp. <10^4^ CFU/ml | *Staphylococcus epidermidis & Streptococcus* spp. <10^3^ CFU/ml | *Staphylococcus epidermidis* 5.500 CFU/ml |
| 82 | *Staphylococcus epidermidis* <10^3^ CFU/ml | *Streptococcus* spp. <10^3^ CFU/ml | Ø |
| 84 | *Staphylococcus epidermidis* <10^3^ CFU/ml | *Staphylococcus epidermidis* <10^3^ CFU/ml | Ø |
| 85 | *Enterococcus faecalis* <10^4^ CFU/ml | *Enterococcus faecalis* <10^3^ CFU/ml | *Staphylococcus epidermidis* 7.700 CFU/ml |
| 90 | *Escherichia coli* <10^3^ CFU/ml | *Escherichia coli* <10^3^ CFU/ml | Ø |
| 91 | *Escherichia coli & Streptococcus* spp. <10^3^ CFU/ml | *Staphylococcus epidermidis & Streptococcus* spp. <10^3^ CFU/ml | *Streptococcus* spp. 1.100 CFU/ml |
| 95 | *Staphylococcus hominis & Streptococcus* spp. <10^4^ CFU/ml | Ø | Ø |
| 97 | *Fusobacterium nucleatum* (16S rDNA) | *Fusobacterium nucleatum* (16S rDNA) | Ø |
| 99 | *Staphylococcus epidermidis* <10^3^ CFU/ml | Ø | Ø |
| 100 | *Streptococcus* spp. <10^3^ CFU/ml | *Streptococcus* spp. <10^3^ CFU/ml | Ø |
| 103 | *Escherichia coli* <10^3^ CFU/ml | Ø | Ø |
| 104 | Ø | Ø | *Streptococcus* spp. 3.330 CFU/ml |
| 105 | *Streptococcus* spp. <10^3^ CFU/ml | *Streptococcus* spp. <10^3^ CFU/ml | *Streptococcus* spp. 1.320 CFU/ml |

Supplementary Table SII Testicular inflammation in association with other inflammation/infection parameters

| Parameter | Testicular inflammation (n=16) | No testicular inflammation (n=55) | p |
| --- | --- | --- | --- |
| Lifetime sexual partners, median (ICR)^a^ | 10 (5-20) | 4 (2-10) | **0.037** |
| History of urogenital tract infection/inflammation | 1/16 (6%) | 4/55 (7%) | 1.000 |
| Amount of leukocytes in VB1 (cells/hpf)^b^ | 0.5 (0.0-1.0) | 0.5 (0.0-1.0) | 0.538 |
| Amount of leukocytes in VB3 (cells/hpf)^b^ | 0.5 (0-1.5) | 0.5 (0.5-0.5) | 0.746 |
| Leukocyturia >20 cells/hpf^c^ | 0/15 (0%) | 1/50 (2%) | 1.000 |
| Peroxidase-positive Leukocytes median (ICR) in 10^6^/ml | 0.1 (0-0.35) | 0.0 (0-0.1) | 0.075 |
| Leukocytospermia (≥ 10^6^/ml) | 1/16 (6%) | 3/55 (6%) | 1.000 |
| Elastase, median (ICR) in ng/ml^d^ | 70 (35-165) | 46 (17-154) | 0.352 |
| Elastase >250 ng/ml^d^ | 3/16 (19%) | 8/54 (15%) | 0.705 |
| Relevant inflammation in urine/semen (Leukocytospermia, VB3 >20 cells/hpf, Elastase >250 ng/ml) | 3/16 (19%) | 8/55 (15%) | 0.702 |
| Presence of bacteriospermia (≥ 10³ CFU/ml) | 6/16 (38%) | 24/55 (44%) | 0.777 |
| Amount of pathogens in bacteriospermia (CFU/ml) | 0 (0-6325) | 0 (0-4400) | 0.725 |
| Presence of relevant bacteriospermia^e^ | 2/16 (13%) | 8/55 (15%) | 1.000 |
| Presence of STIs in urogenital tract | 3/16 (19%) | 5/55 (9%) | 0.368 |
| Presence of pathogens >10^5^ CFU/ml in urine specimens | 1/16 (6%) | 2/55 (4%) | 0.541 |
| Presence of pathogens in swabs/testicular tissue | 0/16 (0%) | 1/55 (2%) | 1.000 |
| All pathogens in urogenital tract | 8/16 (50%) | 25/55 (46%) | 0.782 |
| Clinically relevant pathogens in urogenital tract | 6/16 (38%) | 13/55 (24%) | 0.339 |
| Fulfilled MAGI definition | 3/16 (19%) | 9/55 (16%) | 1.000 |
| NIH-CPSI-Score total score^f^ | 1 (0-8) | 2 (0-5) | 0.667 |

^a^n=68

^b^n=62

^c^n=65

^d^n=70

^e^Urethral commensals (Supplemental Table 1) excluded

^f^n=55

hpf = high power field

Supplementary Table SIII Association of standard clinical parameters and testicular sperm retrieval

| **Parameter**^a^ | **OA (n=15)** | **NOA pos (n=26)** | **NOA neg (n=30)** | **p** |
| --- | --- | --- | --- | --- |
| **Demographics** |  |  |  |  |
| Patient's age (years) | 34.0 (30.0-39.0) | 33.5 (30.8-37.3) | 33.5 (29.0-38.0) | 0.776 |
| Body size (cm) | 176 (172-182) | 180 (175-183) | 180 (175-185) | 0.233 |
| BMI (kg/m²) | 24.7 (22.8-26.1) | 25.1 (23.4-29.1) | 27.0 (24.0-29.6) | 0.310 |
| Body weight (kg) | 78 (72-85) | 81 (74-95) | 87 (79-96) | 0.172 |
| **Risk factors** |  |  |  |  |
| Risk factor for azoospermia (genetics, history UTI, cryptorchidism, cancer) | 4/15 (27%) | 13/26 (50%) | 19/30 (63%) | 0.068 |
| **Semen parameters** |  |  |  |  |
| Glucosidase (mU/ejaculate) | 19.2 (11.4-29.8) | 28.9 (17.1-53.5) | 40.7 (19.0-56.0) | **0.043** |
| **Hormones** |  |  |  |  |
| FSH (mU/ml) | 4.2 (2.7-7.6) | 20.4 (16.5-26.6) | 28.7 (17.6-39.2) | **0.000** |
| LH (mU/ml) | 3.2 (2.0-4.0) | 7.9 (5.3-13.0) | 8.8 (6.0-17.6) | **0.000** |
| Testosterone (nmol/l) | 14.6 (11.6-17.5) | 13.7 (9.7-15.4) | 10.5 (7.8-14.8) | 0.094 |
| Free testosterone (pmol/l) | 290.4 (203.7-322.7) | 248.8 (205.1-325.5) | 229.9 (187.5-272.7) | 0.205 |
| SHBG (nmol/l) | 30.9 (22.8-45.8) | 32.2 (25.9-42.6) | 27.3 (20.9-38.5) | 0.259 |
| Albumin (g/l) | 46.6 (44.7-48.1) | 47.3 (46.0-49.0) | 48.1 (46.4-49.5) | 0.372 |
| Estradiol (pmol/l) | 99.1 (73.4-135.8) | 123.0 (84.4-143.2) | 104.6 (90.9-130.3) | 0.723 |
| Prolactin (uIU/ml) | 166.0 (123.0-188.0) | 146.0 (118.0-213.3) | 167.5 (122.8-217.3) | 0.806 |
| **Ultrasound** |  |  |  |  |
| Total testicular volume (ml)^b^ | 23.1 (18.4-31.2) | 13.2 (7.5-16.3) | 12.0 (4.6-16.8) | **0.000** |
| Mean testicular volume (ml)^b^ | 14.7 (11.3-15.6) | 6.6 (3.8-8.2) | 6.7 (2.8-8.4) | **0.000** |
| Epididymal head height (mm)^c^ | 12.3 (10.5-15.0) | 9.8 (7.9-11.2) | 8.7 (7.3-10.5) | **0.000** |
| Epididymal head thickness (mm)^c^ | 9.4 (7.8-11.3) | 7.2 (6.0-8.9) | 8.0 (6.7-9.7) | **0.029** |
| PSV testicular artery (cm/sec)^b^ | 8.3 (6.9-9.3) | 5.8 (5.0-7.5) | 6.7 (5.8-7.4) | **0.033** |
| PSV intratesticular arteries (cm/sec)^b^ | 4.7 (4.4-6.1) | 4.1 (3.4-5.2) | 3.9 (3.3-4.8) | **0.019** |

^a^data presented as median (IQR), or n (%)

^b^6 patients with single testis excluded

^c^7 patients with single epididymis excluded

PSV = Peak Systolic Velocity
